# Is Alcohol Consumption Related to Lifestyle Factors in Romanian University Students?

**DOI:** 10.3390/ijerph18041835

**Published:** 2021-02-13

**Authors:** Bogdana Adriana Năsui, Rodica Ana Ungur, Patricia Talaba, Valentin Nicolae Varlas, Nina Ciuciuc, Cristina Alina Silaghi, Horatiu Silaghi, Dana Opre, Anca Lucia Pop

**Affiliations:** 1 Department of Community Health, “Iuliu Hațieganu” University of Medicine and Pharmacy, 6 Louis Pasteur Street, 400349 Cluj-Napoca, Romania; adriana.nasui@umfcluj.ro (B.A.N.); talabapatricia94@gmail.com (P.T.); 2 Department of Rehabilitation, Faculty of General Medicine, “Iuliu-Hațieganu” University of Medicine and Pharmacy, 8 Victor Babeș Street, 400012 Cluj-Napoca, Romania; rodica.ungur@umfcluj.ro; 3 Department of Obstetrics and Gynaecology, Filantropia Clinical Hospital, 11171 Bucharest, Romania; 4 Department of Endocrinology, “Iuliu Hatieganu” University of Medicine and Pharmacy Cluj-Napoca, Victor Babes Street 8, 400012 Cluj-Napoca, Romania; alina.silaghi@umfcluj.ro; 5 Department of Surgery V, “Iuliu Hatieganu” University of Medicine and Pharmacy Cluj-Napoca, Victor Babes Street 8, 400012 Cluj-Napoca, Romania; horatiu.silaghi@umfcluj.ro; 6 Department of Educational Science, University Babes Bolyai, 1-3 Kogalniceanu Street, 400084 Cluj-Napoca, Romania; dana.opre@ubbcluj.ro; 7 Department of Clinical Laboratory, Food Safety, “Carol Davila” University of Medicine and Pharmacy, 6 Traian Vuia Street, 020945 Bucharest, Romania; anca.pop@umfcd.ro

**Keywords:** alcohol consumption, students, lifestyle, sleep, nutrition, Romania, Europe, smoking, illicit drugs

## Abstract

Poor eating habits and substance abuse are major public health concerns among young adults transitioning into university life. This study’s objective was to assess Romanian students’ alcohol consumption and correlate it with other lifestyle factors. We performed a cross-sectional study of 1212 students with a mean age of 21.1 ± 2.4 years. We applied a valid online questionnaire to evaluate and statistically analyze the interrelation between alcohol consumption and lifestyle factors by multivariate statistical analysis. The prevalence of alcohol consumption among the student population enrolled in the study was 79.9%. Multiple regression showed that alcohol consumption was positively associated with gender (*p* < 0.001), level of physical activity (*p* = 0.009), number of cigarettes (*p* < 0.001), and fast-food consumption (*p* < 0.001), and negatively associated with sleep (*p* = 0.012) and study hours (*p* < 0.001). The study revealed a high prevalence (18%) of binge drinking among males. The frequent use of illicit drugs is associated with alcohol consumption (*p* < 0.001) but present at low levels (1.6%). The study evidenced a high prevalence of alcohol consumption in students, especially in males, and poor food behavior related to the intake of vegetables and fruits. Health promotion campaigns regarding the harmful effects of alcohol, smoking, poor nutrition, and ongoing illicit drug prevention campaigns are needed to improve students’ performances.

## 1. Introduction

Most non-communicable diseases are linked to common risk factors, namely, tobacco use, harmful alcohol use, an unhealthy diet, and lack of physical activity (WHO). Non-communicable diseases, including heart diseases, diabetes, strokes, cancers, and chronic lung diseases, are responsible for almost 70% of all deaths worldwide [1]. A great proportion of these diseases are preventable by modifying lifestyle factors. Alcohol consumption contributes to millions of deaths each year globally and to the disabilities and poor health of millions of people [2].

According to the 2016 World Health Organization Global status report on alcohol and health, about 3 million deaths per year worldwide (5.3% of global deaths) were attributable to harmful use of alcohol, and 28.7% of the global burden linked to alcohol consumption worldwide in 2016, were due to injuries [2].

Economic and political changes in Romania in recent decades have had a significant impact on different social groups. Young people are undoubtedly a vulnerable group and open to all novelties and changes. University students report exciting, animating, and empowering experiences throughout their university lives, but these are coupled with stressful periods due to academic workload, the pressure to succeed, and competition among peers [3].

Poor eating habits and substance abuse are major public health concerns among young adults who experienced the transition into university life, during which they are exposed to stress and lack of time [4]. Although these behaviors are considered temporary as part of university life, they may persist late in adult life.

In particular, students are at risk for substance abuse behaviors because of lifestyle changes, reduced parental support, and stress [5]. Alcohol remains the number one substance abuse problem throughout university life [6]. The developmental need to form new peer relationships is prominent during this period and is correlated with successful adaption [7]. Alcohol is an important social lubricant in the student population and may be influential due to its role in facilitating social group formation, which may increase the risk of exposure to heavy drinking. Binge drinking (B.D.) is associated with smoking and illicit drug use among students [8]. Heavy drinking is also associated with multiple adverse outcomes in the student population, such as accidents [9], being a victim of a crime, and an increased risk of unprotected sex [10].

Among college students, the prevalence of 30-day alcohol use by college-age females is now equivalent to or exceeding that of their male counterparts. Additionally, the prevalence of high-risk alcohol use among college women has been rising since the 1990s, so that we now see virtually the same proportion of males and females reporting binge drinking on a typical drinking occasion [11]. By exceeding weekly limits more often than men, women put themselves at increased risk for experiencing such long-term effects as liver disease and breast cancer [12]. According to the European School Survey Project on Alcohol and Other Drugs (ESPAD) report in Romania, there are large gender differences regarding alcohol consumption, with higher rates for males than females (differences of more than ten percentage points) and binge drinking more prevalent among males than females [13]. The currently available survey data reveal that B.D. is a prevalent alcohol consumption pattern [14]; less is known about the Romanian student population’s excess drinking habits among males and females correlated with lifestyle factors, such as other risky behaviors, food behaviors, physical activity, and sleep behaviors [15].

Given that alcohol is a modifiable factor, identifying alcohol consumption determinants is valuable for developing prevention programs to reduce harmful alcohol consumption and alcohol-related issues and protect young adults.

Thus, our study aimed to characterize lifestyle factors that influence Romanian university students’ alcohol consumption, particularly related to intake and binge drinking frequency. In addition to alcohol consumption, we evaluated other risky behaviors among university students related to alcohol intake. Another objective was to estimate alcohol consumption among genders and the association between intake and certain demographic and health-related lifestyle factors.

## 2. Materials and Methods

### 2.1. Study Design and Population

We conducted a cross-sectional study based on a valid structured questionnaire conducted online among Romanian University students (males and females) during the 2018–2019 academic year (1 October 2018–31 July 2019).

We used a multistage cluster sampling method to draw a representative sample of 1212 university students. We developed the study in Cluj, from the north-west part of the country, the fourth biggest county in the country, and the second biggest university town in the country after the capital, Bucharest. The Cluj area comprises about 706,905 inhabitants—of which 63,000 students of 377,000 from all over the country [16]- and is divided into smaller urban and rural administrative units (clusters). In the first stage of the study, from six universities in Cluj (based on data from the education management information system of the Centre of Information Technologies in Education), we generated two random numbers between one and six correspondings to the Medical University and the Technical University, representing 36% of the total students in the studied area.

**Figure 1:**
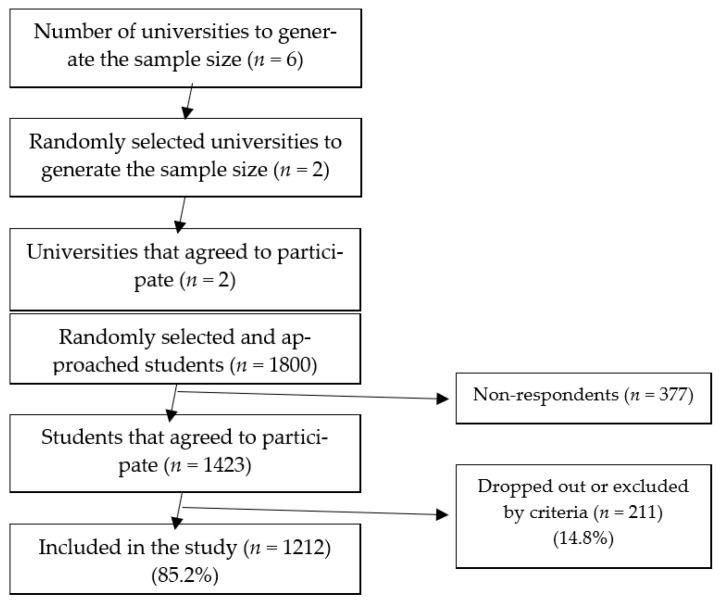
Reporting flow diagram (guideline).

The two universities agreed to participate in the study (a block). Of these, we randomly selected and approached 1800 students (900 per university, from 1 October to 31 July 2018), from which 1423 students agreed to complete the study (Figure 1). The subjects were asked to complete an online valid structured questionnaire during the given period.

The informed consent was given by completing the online questionnaire. The Ethical Committee approved the study protocol of “Iuliu Hatieganu University” (No. 45.02/2019).

We calculated the sample size using Paniott’s formula with an error of 5% based on the total student population; this amounted to 368 participants according to the Romanian Statistics Institute. We calculated a computed minimal size of 659 participants for a 99% confidence level, with a 5% margin of error. Considering a response rate of about 75% (n = 1350), we invited 1800 participants to the study.

We collected data from 1212 students who met the inclusion criteria, voluntarily agreed to participate in the study, and were informed about the aim and procedure.

#### Inclusion Criteria

Inclusion criteria were the following: students enrolled on a degree in one of the universities during the academic year 2018–2019, aged between 18–30 years, and without declared medical conditions—exclusion criteria: students over 30 years old.

### 2.2. Data Collection

(1) The research team developed the questionnaire based on a pretested, valid lifestyle questionnaire. The questionnaire was pretested and re-tested on a sample of 30 subjects. Spearman’s correlation coefficient was used to assess the reliability (r = 0.763).

The time necessary to fill in the survey questionnaire was 25 min. The questionnaires were anonymous and filled on a volunteer basis. Questionnaires were auto-administered and fulfilled by email. Each questionnaire included an embedded, unique respondent number to prevent duplicate responses from the same students or outside the random sample.

The questionnaire included the following:

(1) (a) Demographic data, such as gender, age, and faculty; (b) risky behaviors: alcohol drinking, smoking, and use of illicit substances; (c) nutrition habits; (d) sleep habits; (e) physical activity; (f) students’ learning habits; (g) wellbeing estimate.
(2) We questioned the frequency and amount of alcohol intake: (a) frequency of intake (never, a few times per month, 1–4 times per week, 5–6 times per week, every day); (b) amount of alcohol intake in terms of excessive alcohol consumption—defined as having consumed at least or more than five drinks in a single sitting within two hours, in the past 30 days (same measure for males and females).
(3) A drink was considered as 40–50 mL spirit or 150 mL wine of 12% alcohol or 500 mL beer (5% alcohol) [13].
(4) Smoking was assessed as frequency and as quantity (number of cigarettes). Illicit drug use was assessed by frequency (never, sometimes, frequent).
(5) Food behaviors were assessed as frequency (never, a few times per month, 1–3 times per week, 4–6 times per week, every day). We included fast food, energy drinks, sugar-sweetened beverages (S.S.B.s), and vegetable and fruit consumption in the food category. In the case of vegetables and fruits, we investigated how many portions were eaten per day. A portion of fruits or vegetables was considered 80 g of a medium-size fruit or a 1/2 cup of chopped/cooked vegetables, 30 g of dried fruits, or 150 mL of fruit or vegetable juice
(6) The questionnaire estimated other healthy behaviors. We included sleep habits and physical activity as healthy behaviors.
(7) Sleep habits were evaluated as (a) proper sleep for 6–8 h on more than three days in the previous week [17] and (b) the amount of sleep per night during the exam period and outside the exam period.
(8) Physical activity was estimated as frequency and quantity (starting from 30 min walking) or only by frequency ("how often do you perform other physical activities like swimming, cycling, running, gym, per week?").
(9) The questionnaire assessed the number of study hours during the exam period and outside the exam period; the questions included the categories 0–2 h, 2–4 h, 4–6 h, and more than 6 h.
(10) The general wellbeing of students was estimated by their stress perception.

### 2.3. Statistics

We performed logistic regression analyses to determine alcohol consumption predictors: age, gender, faculty, academic year, sleeping habits, food behaviors, smoking, illicit drug consumption, physical activity, and stress. The tested model explained 25.8% of the variance of alcohol consumption (F = 18, 1180, *p* < 0.0001).

We performed the statistical analysis using the I.B.M. Statistical Package for Social Sciences (SPSS) version 20 (SPSS Inc.^®^, Chicago, IL, USA) and Excel (Microsoft Office^®^ 2010, Albuquerque, NM, USA). We performed the randomization using the Random Number Generators function in SPSS. We described the continuous variables using the mean and standard deviation with a 95% CI. We used cross-tabulations, Pearson chi-squared tests, and logistic regression modeling to create descriptive statistics and test hypotheses.

Results with *p* < 0.05 were considered statistically significant.

## 3. Results

### 3.1. Demographics of the Studied Group

The sample consisted of 58.8% females (*n* = 713) and 41.2% males (*n* = 499). The studied sample’s mean age was 21.1 ± 2.4 years, between 18 and 30 years old. The mean age for females was 21.6 ± 1.8 years, and the mean age for males was 21.7 ± 4.5. Most students (32%) were in their second academic year, the rest being in their first (17%), third (18%), fourth (19%), fifth (11%), or sixth year (3%).

### 3.2. Risky Behaviors among Study Group—Alcohol Habits, Illicit Drug Use, and Tobacco Smoking

#### 3.2.1. Alcohol Habits in the Study Group

**Figure 2:**
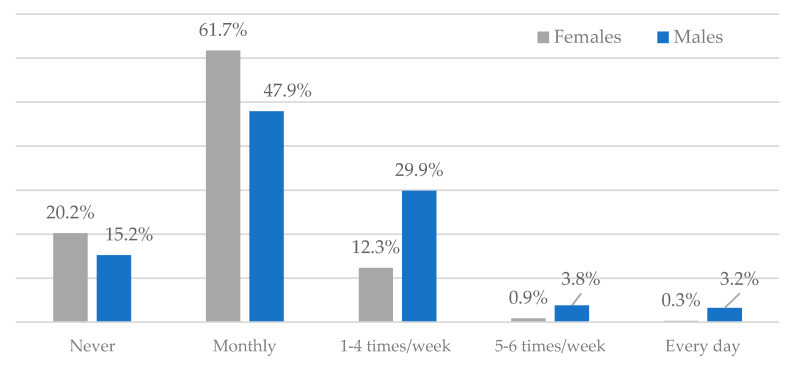
Risky behaviors: alcohol habits by frequency of intake and by gender.

Regarding the frequency of intake by gender, the study emphasized a higher prevalence of alcohol consumption in male students (84.8%) than female students (79.8%). However, most males consume alcohol more frequently—1–4 times per week (29.9%) (Figure 2).

**Figure 3:**
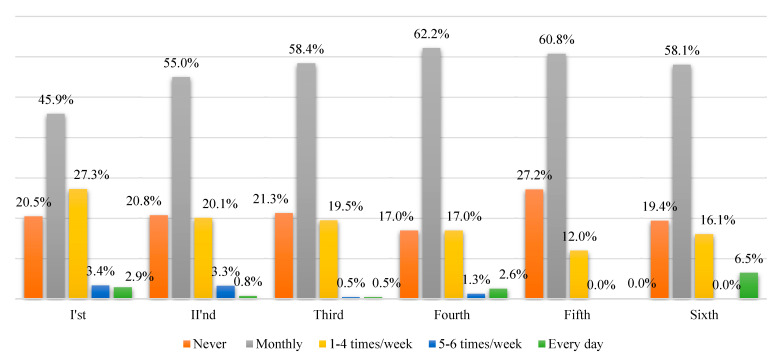
Risky behaviors: alcohol habits by frequency of intake per study year (*p* < 0.001).

In our study, we investigated alcohol consumption during the progression through the academic study years. Regardless of the academic year, most students drink alcohol monthly; in the first year, the students drink alcohol 1–4 times per week in the highest percentage, compared to their colleagues in upper study years. Surprisingly, in the fifth year, alcohol consumption is reduced, and in the sixth year, there is a greater percentage of students (6.5%) that consume alcohol daily (Figure 3).

**Figure 4:**
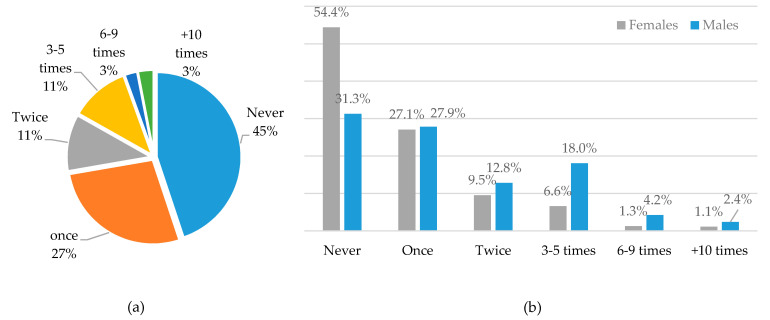
Frequency of excessive alcohol consumption—binge drinking (>5 drinks in one drinking session) per month (**a**) in the total sample (**b**) by gender.

Regarding the amount of intake in terms of excessive alcohol consumption, consisting of more than five standard drinks per month, 45% of students do not consume alcohol at all; meanwhile, 3% (*n* = 37) consume five standard drinks at once (binge drinking episode) (Figure 4a) more than ten times per month, of which 2.4% are males (*n* = 29) and 1.1% are females (*n* = 8) (Figure 4b). A high percentage of males (18%, *n* = 90) are excessive drinkers, consuming more than five drinks in a row 3-5 times per month (18.0%) (Figure 4b).

#### 3.2.2. Other Risky Behaviors in the Study Group—Illicit Drug Use and Tobacco Smoking

Our study showed that most of the students in our study group have never used illicit drugs, 87.4% of females and 76.6% of males, *p* < 0.001 (Table 1). The prevalence of smokers was about 45.5% in the studied sample, higher in females than in male students. Our study’s results evidenced a higher percentage of current male smokers, 26.25%, and 19.2% female smokers.

### 3.3. Food Behavior among the Study Group

Regarding food behavior, we analyzed the intake of vegetables and fruits, fast food, sugar-sweetened beverages (sodas), and energy drinks. The present study showed that students, both females, and males, ate a suboptimal amount (one or two portions) of vegetables and fruits daily (34.3%). Male students consumed fast food more often (1–3 times per week) than girls (*p* < 0.001). Our study evidenced that 22.8% of the male students ingested sugar-sweetened beverages (S.S.B.s) more regularly (1–3 times per week) than girls. The males preferred the energy drinks compared to girls (*p* < 0.001); overall, we found statistically significant differences between males and females in terms of food behaviors—young males having less healthy food habits than similar age females (*p* < 0.001) (Table 2).

### 3.4. Healthy Behaviors in the Study Group—Physical Activity and Sleep Duration

#### 3.4.1. Physical Activities

In total, 60.31% (*n* = 731) of Romanian students walked every day for at least 30 min, with a statistically significant higher prevalence in males (*p* < 0.001). In addition to walking, 26.6% (*n* = 132) of male and 25.7% (*n* = 183) of female students performed other physical activities 1–3 times per week (Table 3).

#### 3.4.2. Sleep Duration

Outside the exam period, most of the students (71.5%, *n* = 867) slept 6–8 h per night, and 22.5% of students (*n* = 273) slept for under six hours; during the period, the percentage of the students with a short sleep duration almost doubled (44.3% vs. 22.5%), mostly in females (*p* < 0.01) (Table 3).

### 3.5. Study Hours per Day

Regarding the time allotted to meet academic assignments, the present study showed that female students studied more than males, both during and outside the exam period. Half of the students (50.4%, n = 611) studied 0–2 h per day outside the exam period. During the exam period, the majority of the students studied over six hours per day (62.6%, *n* = 759), significantly higher in the females than in the males (*p* < 0.001) (Table 4).

### 3.6. Factors Associated with Alcohol Consumption According to Multivariate Logistic Regression

We performed a multivariate logistic regression in order to determine the predictors of alcohol consumption among Romanian students. Our study evidenced that alcohol consumption is directly associated with gender (males) (*p* < 0001), illicit drug use (*p* < 0.001), number of cigarettes (*p* < 0.001), and physical activity (*p* = 0.009). Additionally, the study revealed that students that consumed alcohol ate fast food the most frequently. According to our results, alcohol intake was indirectly associated with sleep (*p* = 0.012); getting enough sleep will reduce alcohol consumption (*p*) and students’ stress (*p* = 0.001) (Table 5).

## 4. Discussion

Poor eating habits and substance abuse are major public health concerns among young adults transitioning into university life. This study’s objective was to assess the alcohol consumption among university students in a representative area of Romania, Europe, and correlate it with other lifestyle factors in order to devise an educational approach to battling the effects of substance abuse in university students, which also becomes a vector of health promotion in family and professional environments. Students have been reported to drink at higher levels than non-student peers, given the negative social and health consequences of excessive alcohol intake and the link with other unhealthy behaviors (e.g., cigarette smoking and recreational drug use) [18]. Previous studies have shown that factors influencing students’ drinking are unstructured time, living situation (on campus, off-campus, and peers), moving away from home, and university life [19,20].

### 4.1. Alcohol and Illicit Drug Abuse

Alcohol and illicit drug abuse in university students is an emerging social and health issue. In the present study, 79.9% of the surveyed students reported alcohol consumption; the prevalence of alcohol consumption was higher among male students than females (84.8% vs. 79.8%). A previous study conducted in the same university town showed that the prevalence was quite similar (90% among males versus 82% among females) [21].

The present study revealed a high prevalence of excessive alcohol consumption, defined by more than five drinks (binge) at least once in the previous month among students, both in males and females (68.7% vs. 45.6%). In addition, 18.0% of male students (n = 90) reported 3–5 binge drinking episodes per month compared to 6.6% (n = 47) females. These results are consistent with other studies that have shown that males are especially engaged in risky alcohol consumption [22,23].

We observed that in the initial academic year, the study sample showed a statistically higher alcohol consumption weekly (1–4 times/week) (27.3%) with a relatively high 5–6 times/week and everyday alcohol consumption (3.4% and 2.9%, respectively), probably due to peer influences [24]. In the following years, the frequent alcohol consumption habit (more than 1–4 times per week) decreased, probably due to high academic assignments; however, in the sixth year, the study reveals the highest daily alcohol consumption (6.5%), suggesting a possible progressively developed increased addictive behavior due to environmental factors during university years. Future studies need to investigate the cause of high alcohol consumption frequencies during the first and final years.

Many people drink for social acceptance because of peer group pressures and to gain adult status and an image of strength, especially men.

Hazardous alcohol consumption is ranked above illicit drug use, causing harm to our society, economics, and health.

The prevalence of illicit substances among Romania’s adult population remains low compared with other countries [25]. Data from a general population study indicate that only 2.5% of young adults tried a new psychoactive substance at least once in their lives. The use was more frequent among males and concentrated among young people of 15–34 years old. In the present study, 2.8% of male students reported frequent illicit drug consumption, and 20.6 % used it a few times. Despite the low drug consumption, the logistic regression showed that Romanian students’ alcohol consumption was directly associated with illicit drug use (*p* = 0.003).

Alcohol and illicit drug consumption may affect school-related problems, such as poor grade achievement and high school absence [26]. In our study, alcohol consumption was inversely associated with the number of study hours during the semester, meaning that those engaged in these risky behaviors are studying less and possibly may have lower grades than others, but this fact was not the aim of our study. Previous studies performed on Romanian students showed that at-risk drinkers had lower grades than low-risk drinkers [19].

Alcohol and drug use and smoking, the substances most abused by the students, are important risk factors for early death: consequently, 11.4 million people die prematurely each year. Data from the literature showed that certain substances are used to cope with stress [27]. Similar to other studies [28], in our studied sample, the prevalence of smoking was high (45.6%), everyday smoking being higher in male students compared to females (26.3% vs. 19.2%); the intensity of smoking (reflected by the daily number of cigarettes) was associated with alcohol consumption, as shown in the multivariate logistic regression.

According to our study results, alcohol consumption is associated with the male gender and surprisingly inversely associated with stress (*p* = 0.001). Students were possibly involved in performing their assignments rather than socializing and consuming alcohol. Another reason for a negative correlation between stress and alcohol is that students cannot focus and concentrate when drunk. Additionally, during the exam session’s period, there might be fewer peer-associated opportunities for getting drunk when the students are learning.

On the other hand, the inverse relationship between alcohol consumption and stress points the fact that the less the students are stressed, the more they consume alcohol, possibly due to an incorrect perception of alcohol as an entertainment tool, a direction that may be further investigated and tackled in public health measures.

### 4.2. Food Behaviors

Even if vegetables and fruits are not linked to alcohol consumption, the study evidenced another unhealthy behavior among female and male students, mostly consuming only one or two portions per day (74.7%). Additionally, many male students (15.4%) reported that they do not eat vegetables and fruits at all. Our results are similar to those of other studies that have revealed that university students fail to meet the recommended intake of fruits and vegetables [29], linked to the future development of digestive and non-digestive cancer pathology [30].

Fast food intake is associated with alcohol consumption. Like previous studies, the present study evidenced that males (34.3%) choose fast food meals in a higher frequency (1–3 times per week). Fast food is energy-dense, low nutritional value food, very popular among students, leading to obesity [31,32]. In Romania, even though healthy food is available on campus and off-campus, and the population’s socioeconomic status is good, study shows that a segment of students choose to eat unhealthy food, often when socializing with their peers, which is a reason for nutrition education in university students.

The most common factors reported as barriers to a healthy diet are time constraints, the high price of food items, and the lack of food preparation motivation [33]. Studies from the literature evidenced that alcohol and eating behaviors are linked in different ways [34]. Cost, resources, and access were continually cited as factors that influence both food and alcohol choices, and young adults reported finding it difficult at times to source healthy food options, particularly after consuming alcohol.

Previous studies conducted on Romanian adolescents have shown an increased intake of processed food, especially sweetened beverages [35]. Unsurprisingly, the present study showed the same results and revealed a high frequency of soda and energy drink intake (especially among males), not associated with the multivariate model’s alcohol consumption. Nevertheless, many students (20.0% more females than males) did not consume sweetened beverages at all.

Energy drinks have been very popular among students in recent decades. Mixing alcohol with energy drinks has been associated with an increase in alcohol consumption, possibly through the effect of masking subjective intoxication [36]. In the present study, Romanian students reported a low consumption of energy drinks. The results showed that alcohol consumption was not significant associated with energy drink intake (*p* = 0.879).

### 4.3. Healthy Behaviors

#### 4.3.1. Physical Activity Related to Alcohol Consumption

Physical activity (P.A.) has been described to reduce the association between alcohol consumption and risk of death [37], whereas others have found an association between higher P.A. and high levels of alcohol consumption [38,39]. Research indicates that college students who engage in regular vigorous physical activity are more likely to participate in social gatherings and show a higher acceptance for alcohol consumption [40]. Like other studies, in our study, alcohol consumption was directly associated with the amount of physical activity.

According to the World Health Organization (WHO), the recommended level of physical activity for adults (18–64 years) is at least 150 min of moderate activity and 75 min of vigorous physical activity throughout the week. These activities may be part of leisure time, transportation, occupational, games, plays, sports, or planned exercises [41].

In the present study, only 60% of students engage in a physical activity every day, and about 26% engage in other physical activity 1–3 times per week. Like other studies, our study showed that male students performed physical activity more frequently than females [42]. According to our study, Romanian students did not meet the physical activity recommendation for a healthy lifestyle to prevent chronic disease (however, one of our study’s limitations was that weekly physical activity was not quantified as time). Previous studies conducted on Romanian students have evidenced that both males and females meet the recommendations [39]. Further studies that will imply physical activity measuring devices will give objective data on students’ physical activities.

#### 4.3.2. Sleep Quality

Healthy sleep is important for both physical and mental health, improving productivity and overall quality of life. Good sleep quality is necessary for good academic performance [43].

Sleep quality is a high prevalence problem among university students. Studies from the literature showed an inverse association between higher insomnia symptoms and illicit drug abuse; good sleep quality is a protective factor against problematic illicit drug use [44]. Alcohol and illicit drug use are positively associated with sleep problems in a dose-dependent manner. Alcohol has a sedating effect on moderate consumption (2–3 drinks). With increasing amounts of alcohol, sleep latency generally decreases [45]. In our study, the number of sleep hours was indirectly associated with alcohol consumption; the more they drank, the less they slept. Students engaged in excessive drinking may have poor quality and duration of sleep, as previous studies reported; otherwise, sleep disturbances are not present in the general sample of the student population.

Alcohol is known to alter sleep and regularity. Alcohol intoxication may lead to a shorter sleep onset latency but poorer sleep quality in the latter part of the sleep, resulting in shorter sleep duration and sleep disruption; in addition, irregularity in sleep-wake schedules are associated with a higher risk of alcohol use [46,47]. The present study shows that alcohol consumption among students is indirectly associated with proper sleep. As the study results showed, these students, especially males, may be engaged in excessive drinking that probably leads to sleep disturbances.

According to sleep organization recommendations, young adults aged 18–25 need seven to nine hours of sleep per night [48,49]. The present study results evidenced that many female students (45.6%) slept for under six hours during the exam period.

These results are similar to those of other studies, showing that females are poorer sleepers than males, probably due to higher stress and anxiety [50]. On the other hand, other studies have evidenced a higher prevalence of poor sleep quality among males, probably attributed to the high prevalence of addiction, alcohol consumption, and illicit drug use among males [51,52].

The study evidenced a high prevalence of alcohol consumption in students, especially in males, and poor food behavior related to the intake of vegetables and fruits.

Health promotion campaigns regarding the harmful effects of alcohol, smoking, improper nutrition, and ongoing illicit drug prevention campaigns, are needed to improve students’ performances. Health promotion programs must be followed by public campaigns in the student environment, especially among males, for which consuming alcohol is a status-enhancing behavior, while women drinking alcohol are demoted and stigmatized in traditional societies.

## 5. Limitations

The present study has several limitations. Among the limitations is the questionnaire method for data collection, which can lead to subjective reporting. Another limitation of the study was that we did not quantify weekly alcohol consumption in terms of standard drinks, only the frequency of excessive (binge) drinking episodes, and did not use standardized identification tests (i.e., alcohol use disorder) [53].

In many countries, drinking is usually considered an important socializing custom in business, relaxing and improving interpersonal relationships. Although alcohol is well accepted in western societies and sometimes not even regarded as a drug, illicit drugs are not accepted, and this fact can influence the self-administered assessment, eventually leading to an under-reporting of drug consumption. Further studies measuring illicit drug use [54] may objectivize the results of our study.

Since females drink less than men, and our sample comprises 58.8% females, this may generate bias on the gender differences in alcohol consumption.

The gender representativeness of the study was influenced by the sampling method (random selection). In addition, the response bias may have effects on the results. The response bias could have also influenced the validity of the (a) alcohol consumption reports and (b) drinking severity self-assessments - in men. On the other hand, as alcohol consumption in women drinking is stigmatized, under-reporting of their consumption might have been encountered.

Another limitation was that the study did not assess the total weekly duration of physical activity, only the walking time, the other physical activity being evaluated as frequency, and physical activity level.

Another limitation was that the method did not use validated scores to measure stress and sleep, which should be considered in the subsequent studies on the topic.

The representativeness of the selected study sample reflects the general population being statistically the second university town in Romania, emphasized by similar (but scarce) studies [55]; this fact could generate potential selection biases, highlighting the need for a following study at the country level.

## 6. Strengths

Our study is among the most significant studies in our geographical area, analyzing a large representative student group and investigating alcohol consumption and other lifestyle factors that may influence alcohol and other risky behaviors. The present study investigated many nutritional factors that characterize the transition period during students’ lives, giving a larger and more precise picture than other previously published studies.

## 7. Conclusions

Our study evidenced a high prevalence of alcohol consumption and binge drinking among Romanian students, higher in males than females. Alcohol consumption was increased in association with the number of cigarettes, illicit drug use, physical activity, and fast-food consumption, and decreased with stress, proper sleep, and study hours among students.

Our study revealed high alcohol consumption as binge episodes in students—not linked to stress—possibly due to the perception of alcohol intake as a fun habit; public health campaigns regarding the harmful effects of alcohol in students’ environments are needed.

The study revealed a low intake of vegetables and fruits and high consumption of fast food in the students enrolled in the study. Health promotion campaigns should include (a) nutrition education campaigns (i.e., the need for vegetable and fruit intake), (b) active lifestyle campaigns - promoting the physical activity, good sleep patterns, and (c) smoking cessation campaigns. The study revealed low levels of illicit drug consumption among Romanian students; nevertheless, illicit drug preventive measures should be maintained in the student environment. Our analysis supports the need for political healthcare campaigns to battle the causes of alcohol consumption that must include the banning of mass media advertising of alcohol consumption as a fun habit and as a feature of strength, and the limitation of alcohol sales inside and in the vicinity the campus may be introduced in internal university rules.

## Figures and Tables

**Table 1:** Risky behaviors among study group—illicit drug use and tobacco smoking.

Illicit Drugs	Total		Females		Males		*p* *
	**No**	**%**	**No**	**%**	**No**	**%**	
Never	1005	82.9	623	87.4	382	76.6	0.000
Frequent	19	1.6	5	0.7	14	2.8	
Several times	188	15.5	85	11.9	103	20.6	
Smoker							
Never	659	54.4	408	57.2	251	50.3	0.04
Sometimes	159	13.1	97	13.6	62	12.4	
Former smoker	86	7.1	49	6.9	37	7.4	
Quitting	40	3.3	22	3.1	18	3.6	
Every day	268	22.1	137	19.2	131	26.3	
No. of cigarettes							
None	747	61.6	460	64.5	287	57.5	0.04
below 10	306	25.2	191	26.8	115	23.1	
10-20/day	134	11.1	48	6.7	12	2.4	
20-30/day	19	1.6	12	1.7	5	1.002	
Over 30	6	0.5	2	0.3	4	0.8	

* *p*-value shows statistical differences between genders.

**Table 2:** Food behavior in the studied group.

Variable	Total		Females		Males		*p* *
	No	%	No	%	No	%	
Vegetables and fruits							
Never	143	11.8%	67	9.4	76	15.2	0.01
1–2 portions	905	74.7%	540	75.7	365	73.2	
3–4 portions	145	12.0%	93	13.0	52	10.4	
Over 5 portions	19	1.6%	13	1.8	6	1.2	
Fast food							
Never	95	7.8	68	9.5	27	5.4	<0.001
Monthly	754	62.2	486	68.2	268	53.7	
1–3 times/week	303	25.0	132	18.5	171	34.3	
4–6 times/week	41	3.4	23	3.2	18	3.6	
Every day	19	1.60	4	0.6	15	3.0)	
Energy drinks							
Never	735	60.6	480	67.3	255	51.1	<0.001
Monthly	416	34.3	211	29.6	205	41.1	
4-6/week	61	5.0	22	3.1	39	7.8	
Sodas (S.S.B.s)							
Never	243	20.0	173	24.3	70	14.0	<0.001
Monthly	538	44.4	334	46.8	204	40.9	
1–3 times/week	235	19.4	121	17	114	22.8	
4–6 times/week	94	7.8	42	5.9	52	10.4	
Every day	102	8.4	43	6.0	59	11.8	

* *p*-value shows statistical differences between genders.

**Table 3:** Healthy behaviors in the study group—physical activity and sleep duration.

**Healthy Behaviors**	**Total (No/%)**	**Female (No/%)**	**Male (No/%)**	*p* *
Physical activity				
*Walking at least 30 minutes*				
Never	34 (2.81)	24 (3.37)	10 (2.00)	<0.001
1–3 days/week	227 (18.73)	144 (20.19)	83 (16.63)	
4–6 days/week	220 (18.15)	122 (17.11)	98 (19.64)	
everyday	731 (60.31)	423 (59.33)	308 (61.72)	
*Other physical activity*				
Never	283 (23.3)	189 (26.51)	94 (18.84)	
Monthly	448 (37.0)	293 (41.09)	155 (31.06)	<0.001
1–3 days/week	315 (26.0)	183 (25.66)	132 (26.45)	
4–6 days/week	119 (9.8)	37 (5.19)	82 (16.43)	
everyday	47 (3.9)	11 (1.54)	36 (7.21)	
**Daily sleep hours**	**Total (No/%)**	**Female (No/%)**	**Male (No/%)**	***p***
*Outside exam period*				
below 6 h	273 (22.5)	157 (22.02)	116 (23.25)	0.74
6–8 h	867 (71.5)	511 (71.67)	356 (71.34)	
+8 h	72 (5.9)	45 (6.31)	27 (5.41)	
*During the exam period*				
below 6 h	513 (42.3)	325 (45.58)	188 (37.68)	0.01
6–8 h	568 (46.9)	320 (44.88)	248 (49.70)	
+8 h	131 (10.8)	68 (9.54)	63 (12.63)	

* *p*-value shows statistical differences between genders.

**Table 4:** Daily study hours in the study group—outside and during the exam period.

Daily Study Hours	Total (No./%)	Females (No./%)	Males (No./%)	*p* *
*Outside the exam period*				
0–2 h	611 (50.4)	325 (45.58)	286 (57.31)	0.001
2–4 h	401 (33.1)	258 (36.16)	143 (28.66)	
4–6 h	158 (13.0)	101 (14.17)	57 (11.42)	
+6 h	42 (3.5)	29 (4.08)	13 (2.61)	
*During the exam period*				
0–2 h	34 (2.8)	8 (1.12)	26 (5.21)	<0.001
2–4 h	99 (8.2)	36 (5.05)	63 (12.63)	
4–6 h	320 (26.4)	146 (20.48)	174(34.87)	
+6 h	759 (62.6)	523 (73.35)	236 (47.29)	

* *p*-value shows statistical differences between genders.

**Table 5:** Factors influencing alcohol consumption multivariate logistic regression.

Model	Unstandardized Coefficients	Standardized Coefficients	t	*p* Value	Collinearity Statistics	Tolerance	V.I.F.
	B	Std. Error	Beta				
(Constant)	2.354	0.231		10.179	0.000		
Faculty	−0.060	0.049	−0.038	−1.246	0.213	0.666	1.502
Gender	0.224	0.046	0.141	4.874	0.000	0.753	1.329
Age	−0.017	0.010	−0.052	−1.700	0.089	0.682	1.466
Academic year	−0.013	0.018	−0.022	−0.687	0.492	0.623	1.606
Study hours outside the exam period	−0.121	0.026	−0.127	−4.661	0.000	0.845	1.183
Study hours during exam period	−0.021	0.030	−0.020	−0.698	0.485	0.752	1.329
Sleep hours outside the exam period	−0.148	0.045	−0.096	−3.285	0.001	0.735	1.360
Sleep hours during exam period	−0.019	0.033	−0.016	−0.566	0.572	0.809	1.237
Appropriate sleep	−0.117	0.046	−0.073	−2.522	0.012	0.754	1.327
Portion of vegetables and fruits	0.034	0.038	0.024	0.888	0.375	0.881	1.136
Fast food	0.132	0.031	0.122	4.253	0.000	0.768	1.302
Sodas	0.004	0.021	0.005	0.181	0.856	0.694	1.441
Energy drinks	−0.005	0.030	−0.004	−0.152	0.879	0.772	1.295
Coffee every day	0.022	0.015	0.039	1.406	0.160	0.818	1.222
Illicit drugs	0.308	0.050	0.169	6.197	0.000	0.850	1.176
No cigarettes/day	0.225	0.029	0.226	7.639	0.000	0.719	1.390
Physical activity	0.053	0.020	0.071	2.617	0.009	0.849	1.177
Stress	−0.156	0.046	−0.092	-3.370	0.001	0.853	1.173

Dependent variable alcohol consumption, *p* < 0.05, was considered statistically significant. **V.I.F.**—variance inflation factor.

## Abbreviations

PA	Physical activity
B.D.	Binge drinking
C.I.	Confidence Interval
N.S.I.	National Statistics Institute
ESPAD	European School Survey Project on Alcohol and Other Drugs
S.S.B.s	Sugar-sweetened beverages
SPSS	Statistical Package for Social Sciences
WHO	World Health Organization
